# Risk of tuberculosis among Alabama children and adolescents treated with tumor necrosis factor inhibitors: a retrospective study

**DOI:** 10.1186/s12969-017-0207-8

**Published:** 2017-11-09

**Authors:** Matthew L. Stoll, James Aaron Grubbs, Timothy Beukelman, Melissa L. Mannion, Traci W. Jester, Randy Q. Cron, Marilyn J. Crain

**Affiliations:** 1 Department of Pediatrics, University of Alabama at Birmingham, CPP N 210M / 1600 7th Avenue South, Birmingham, AL 35233 USA; 2 Department of Medicine, University of South Carolina School of Medicine, Medical Park, Suite 420, Columbia, SC 29203 USA; 3 Department of Microbiology, University of Alabama at Birmingham, Birmingham, AL USA

**Keywords:** Juvenile idiopathic arthritis, Tumor necrosis factor inhibitors., Tuberculosis.

## Abstract

**Background:**

Tumor Necrosis Factor inhibitors (TNFi) have dramatically improved the outlook for patients with inflammatory arthritides and bowel disease (IBD), but are associated with increased infection risks, including tuberculosis (TB). Pediatric inflammatory diseases are uncommon, and the risk of TB in children taking TNFi remains unclear. The objective of this study was to report the incidence of TB disease among TNFi recipients at a single pediatric medical center serving most of Alabama compared to that of the general population of Alabama children.

**Methods:**

Instances of TNFi usage among patients under age 20 years from July 1, 2007 through April 17, 2015 were captured from electronic health records at Children’s of Alabama (CoA), which has the only pediatric rheumatology clinic in Alabama, and where a substantial number of children in Alabama with inflammatory bowel disease receive care., and reports of TB cases were obtained from the Alabama Department of Public Health (ADPH). Incidence was expressed as TB cases/10,000 person-years, using population estimates from the Alabama Center for Health Statistics.

**Results:**

1033 Alabama patients at CoA who were residents of Alabama were identified who received TNFi for a total of 1564 person-years. One adolescent on TNFi developed severe extrapulmonary TB (incidence density = 6.4 per 10,000; 95% CI 0.9–45.4 per 10,000). Sixty-three cases occurred in persons not on TNFi (incidence density = 0.064 per 10,000; 95% CI 0.050–0.082 per 10,000).

**Conclusions:**

One case of TB disease among TNFi-exposed children was identified for 1564 person-years in Alabama residents. Although rare, this is higher than expected relative to the general rate of TB in Alabama. Thus, continued diagnostic vigilance for TB in children taking TNFi is required.

**Trial registration number:**

Not applicable.

## Background

Tumor Necrosis Factor inhibitors (TNFi) have revolutionized therapy for a variety of autoimmune conditions, including pediatric and adult arthritis and inflammatory bowel disease (IBD) [1]. However, there have been multiple case reports of TB disease among adult and pediatric patients exposed to TNFi [2], as well as a meta-analysis in adults indicating that TNFi exposure was associated with a higher risk of TB [3]. TNF is required for granuloma formation to contain *Mycobacterium tuberculosis*; impaired granuloma formation appears to be responsible for increased risk of disseminated infection [4]. The FDA includes a black box warning for the entire class of TNFi with recommendations for TB testing prior to initiation of TNFi therapy [5].

The risk of TB infection and disease among TNFi recipients is related to opportunities for exposure to infectious TB, either in the community or through travel to a high prevalence area, and receipt of preventive therapy if indicated. The Swedish registry of adults with rheumatoid arthritis (RA) indicated a four-fold higher risk of active TB among TNFi users compared to non-users, compounded on the two-fold higher baseline risk in RA patients compared to the general population [6]. A meta-analysis of infections in pediatric TNFi recipients reported 5 TB cases among 2949 patients with juvenile idiopathic arthritis (JIA) on TNFi and none among 1648 patients with inflammatory bowel disease (IBD), but no incidence rates could be calculated [7]. Thus, in the United States, incidence data for TB in pediatric patients on TNFi is limited. Despite this limited data, recommendations for TB screening in children with JIA were recently switched from annual laboratory-based screening to screening of high-risk subjects [8]. To try to address tuberculosis risks in Alabama children on TNFi, we compared the incidence of TB disease among children and adolescents exposed to TNFi at a single clinical site in Alabama with that of the Alabama pediatric population not exposed to TNFi during the same years of study.

## Methods

### Identification of patients exposed to TNFi

Patients included in the exposure group were Alabama residents 0–19 years of age who had received TNFi prescriptions or had infusion of TNFi at CoA between July 1, 2007 and April 17, 2015 for any indication. Although the older subjects in this cohort are not typically considered pediatric subjects, we chose this age range for purposes of consistency with the Alabama Department of Public Health (ADPH) database. Two sources of information were used to identify children exposed to TNFi in Alabama. First, the electronic medical record (EMR) at CoA was queried for all prescriptions for each of the five TNFi available (adalimumab, certolizumab pegol, etanercept, golimumab, and infliximab) from July 1, 2007 through April 17, 2015. Although infliximab is given as an infusion, its use was typically recorded on the same medication list as used to document prescriptions. Second, CoA billing records were analyzed to identify all infliximab infusions occurring at CoA during the same time period. The combination of these methods allowed for the best capture of patients who received infliximab at CoA or through home health care agencies (for which infliximab use would only be documented through the EMR).

The majority of children in Alabama treated with TNFi receive care at Children’s of Alabama (CoA) and affiliated clinics where the only pediatric rheumatology practice in the state of Alabama is located; additionally, most patients treated with infliximab for IBD in the greater Birmingham area receive their infusions at CoA. Thus, prescribing data are available for the vast majority of instances of pediatric TNFi use in AL (subcutaneous adalimumab and intravenous infusions of infliximab for IBD and JIA, and all five TNFi for JIA).

From billing documents and the EMR, the following information was abstracted: name, data of birth, race, sex, state of residence, diagnosis, specific TNFi medication, and dates of TNFi usage. Exposure periods were censored as of the date when the subject reached 20 years of age in order to be consistent with the ADPH database of reported cases of TB disease. All TNFi recipients were included if they were Alabama residents. Not all instances of TNFi usage are for indications approved by the FDA.

### Identification of TB cases

A list of all children and adolescents 0–19 years of age who were reported as TB cases between July 1, 2007 and April 17, 2015 was obtained from the ADPH Division of TB Control. All cases of active tuberculosis are required by Alabama state law to be reported to ADPH, who subsequently report the cases to the Centers for Disease Control and Prevention. This data is reported in five-year age intervals, e.g. 0–5 years, 5–9 years, etc. For ethical reasons, this list was maintained exclusively by one of the authors (MJC), who is affiliated by contract with the ADPH. MJC was also provided with the list of subjects at CoA exposed to TNFi and was able to identify any person appearing on both lists. The ADPH list of reported TB cases was not shared with other co-authors.

### Statistical analysis

The study period was 7.8 years. To calculate population-based person years, 2010 US Census data and annual intercensal population estimates were used for the state of Alabama from the Alabama Public Health Center for Health Statistics (Alabama Vital Statistics Report Table 85 http://www.alabamapublichealth.gov/healthstats/assets/AVS2014.pdf) for persons aged 0–19 years, inclusive. The population estimate for each year was assigned on July 1 for each year in the study. As no estimates were yet available for the year 2014, the mean of the annual population for the years 2007–2013 (1,258,387, with a standard deviation 10,991) was used to estimate the population on July 1, 2014.

Confidence intervals for the calculated incidence density rate of TB cases in cases per 10,000 person-years in children exposed or unexposed to TNFi were calculated according to the Poisson distribution using SAS (Cary, NC). The incident case rate ratio for TB was then calculated for patients exposed to TNFi compared to the TNFi unexposed population of Alabama children and adolescents.

## Results

During the study period, 1090 subjects were exposed to TNFi at CoA for a total of 1631 person-years, of whom 1033 resided in Alabama (1564 person-years) (Table 1). Many patients (26%) were exposed to multiple agents sequentially, so the sum of patients exposed to the individual agents exceeds the overall number of patients on all TNFi. Fifty-seven percent of TNFi-exposed patients were female, and the racial breakdown was representative of Alabama (75% Caucasian, 24% African-American; Table 2); information on ethnicity was not reliably available through the EMR. JIA and IBD were the most common indications for TNFi use. The median age (interquartile range) at initial TNFi exposure was 13.0 years (9.2–15.8); the youngest child was 18 months. The median duration of exposure per individual was 1.0 years (interquartile range 0.17–2.4).

**Table 1:** Exposure of pediatric patients residing in Alabama to individual TNFi and patient-years of exposure for each^a^

Medication	Number of subjects exposed	Total patient-years of exposure
Adalimumab	469	495.3
Certolizumab pegol	9	2.0
Etanercept	324	194.6
Golimumab	6	1.5
Infliximab	527	840.6
All TNFi	1033	1564.3

^a^Because many subjects were exposed to more than one TNFi, the total number of subjects does not equal the sum of the subjects for the individual medications

**Table 2:** Exposure to any TNFi by pediatric patients at CoA residing in Alabama

Characteristic	Value
n	1033
Age at initial usage (median, IQR)	13.0 (9.2–15.8)
Years of exposure (median, IQR)	1.0 (0.17–2.4)
Female	592 (57%)
Race	
Caucasian	771 (75%)
African-American	251 (24%)
Other / unknown	11 (1.1%)
Number of TNF inhibitors used	
1	768 (74%)
2	227 (22%)
3	37 (3.6%)
4	1 (0.1%)
Diagnosis	
JIA^a^	544
IBD^a^	265
IBD with JIA^a^	69
Vasculitis	33
Uveitis	31
Psoriasis	29
Sarcoidosis	17
Mixed connective tissue disease / SLE	13
Sjogren Syndrome	7
Recurrent fever syndrome	6
Behcet Syndrome	5
Hidradenitis	3
Juvenile dermatomyositis	3
Non-infectious osteomyelitis	3
Sensorineural hearing loss	2
Other^b^	3
Cases of Tuberculosis	1

Abbreviations: *IBD* inflammatory bowel disease, *JIA* Juvenile idiopathic arthritis, *SLE* systemic lupus erythematosus

^a^Includes subjects with uveitis, psoriasis, or both

^b^Includes one each of chronic granuloma annulare, pityriasis rubra pilaris, and orbital pseudotumor

During this time period, we identified a single case of TB disease among the population of TNFi-exposed pediatric patients who resided in Alabama (1 case per 1564 person-years), an incidence rate of 6.4 per 10,000 person-years (95% CI 0.9–45.4 per 10,000 person-years). This patient was a 13-year-old girl with severe Crohn disease (CD) who developed disseminated TB while on treatment with adalimumab and methotrexate. She was diagnosed with CD three and one half years prior to diagnosis of TB and had received nearly continuous treatment with TNFi and methotrexate beginning two months after IBD diagnosis, with infliximab 100 mg infusions every 6 weeks over 22 months, followed by certolizumab pegol 200 mg subcutaneously every month after induction for nine months. Following a break of two months, she was started on adulimubab 20 mg subcutaneously every two weeks after initial induction for three months followed by a dosage increase to 40 mg every two weeks for four months with ongoing methotrexate. She was on varying doses of prednisone for most of the first three years of her course. The patient weighed 24–29 kg over the entire period of TNFi treatment. Two documented tuberculin skin test (TST) results were 0 mm induration. The first was placed four months after diagnosis of CD and two months after initiation of the first TNFi. The second was placed two years later, four months prior to her TB exposure and sixteen months prior to her TB diagnosis; no TST result prior to TNFi initiation could be documented. Initial symptoms of TB began 37 months into her course of CD, prior to initiation of adalimumab therapy, and diagnosis occurred five months after development of symptoms. Disease sites included synovium, brain parenchyma with multiple punctate lesions, lumbosacral diskitis, vertebral osteomyelitis, and a large paraspinal abscess. Cultures from synovium and the paraspinal abscess grew *M. tuberculosis*. QuantiFERON® TB was positive at diagnosis of TB and her TST was positive at 17 mm. With aggressive anti-mycobacterial therapy, cessation of her TNFi therapy, surgical intervention, and interventional radiology procedures, she recovered from her disseminated TB. Her TB exposure had occurred approximately 12 months prior to diagnosis of her TB disease. She was not named as a contact during the epidemiologic investigation of the person with infectious TB to whom she was exposed. She was around this individual only over a short period of time. The genotype of her organism matched that of the putative source case, suggesting that the correct source of her TB infection and disease had been identified. No history of foreign travel or other TB risk factor was identified.

All individuals 0–19 years of age in Alabama during the study period accounted for 9,815,420 person-years; individuals without ongoing TNFi exposure accounted for 9,813,853 person-years. Of the sixty-four cases of TB diagnosed among Alabama children and adolescents 0–19 years of age, 63 cases had not been exposed to TNFi. The incidence density of TB in Alabama’s children and adolescents without TNFi exposure during the years of study was 0.064 per 10,000 person-years (95% CI 0.050–0.082 per 10,000 person-years). The rate ratio for incident TB disease among TNFi-exposed versus TNFi-unexposed pediatric patients in Alabama during the study period was 99.69 (95% CI is 13.82–719.02; *p*-value is <0.0001). However, as not all children in Alabama are routinely screened for TB, the true incidence of TB in children and adolescents without TNFi exposure may be higher.

## Discussion

We identified a single case of TB among TNFi-exposed patients aged 0–19 years in Alabama during our study period. This adolescent had severe, disseminated TB in the context of highly active IBD (a known risk factor for serious infection in IBD) [9]. Similar extent and severity of TB disease and similar delays in TB diagnoses have been reported in other patients on TNFi who develop TB. However 1032 of 1033 patients on TNFi therapy did not develop TB disease, allowing continued treatment for many children suffering from JIA, IBD, and a variety of other disorders. Nevertheless, one child with TB resulted in an increased overall risk for TB in TNFi-exposed as compared to TNFi-unexposed children. This report demonstrates the need for vigilance in TB screening and diagnosis in TNFi-exposed patients, even in a country with a low incidence of TB.

Over the years of this study, the overall incidence of TB disease decreased in the US from 4.4 cases per 100,000 persons in 2007 to 2.9 cases per 100,000 persons in 2015. Similarly the case rate in Alabama over the same years decreased from 3.8 to 2.5 cases per 100,000 persons, respectively. (https://www.cdc.gov/tb/statistics/reports/2015/default.htm, accessed 10/5/2017) Given the comparable overall cases rates between Alabama and the US overall, it seems likely that these Alabama data may be applicable in most other regions of the US as well, even though the age groupings are not completely congruent between our study and the CDC statistics.

Adult registry data have indicated that most cases of TB in persons on TNFi occur early in the course of therapy, consistent with reactivation of previous infection [6]. However TB disease may occur in both children and adults following acquisition of TB infection while on TNFi, as appears to have been the case for our single patient with disseminated TB [10]. Recently, TB was reported in two adolescents treated with TNFi for IBD and whose screening TSTs were negative prior to initiating therapy [2], similar to our case. Thus, while routine annual screening may not identify all cases of TB, it may allow for the detection of some new TB infections prior to their progression to TB disease.

This study, conducted at a single clinical center, has limitations. TB screening test results were not available for TNFi recipients nor for the general pediatric population; thus we cannot compare TB infection rates, only diagnosed and reported TB disease. Our study included only 1564 person-years of TNFi exposure among pediatric patients, a small sample size which limited our power to examine the incidence of a disease that occurred at a rate of 2.96 cases/100000 persons in the United States in 2014. A single case in the TNFi recipient group resulted in a very broad CI around the calculated incidence density of 6.4 per 10,000 person-years (95% CI 0.9–45.4 per 10,000 person-years).

Our capture of patient exposure to TNFi was also subject to limitations. Pediatric patients receiving TNFi could be underreported, especially for IBD where treatment was less centralized than for rheumatologic conditions. Pharmacy claims information was not available. Billing data for infliximab was based upon actual visits and thus provided documentation of use, but did not indicate adherence to administration at home; thus TNFi use could be overestimated. TNFi exposure was relatively brief in duration for 25% of the patient group, at less than two months. It is possible that their inclusion minimized the risks of TB compared to inclusion of only those with longer TNFi exposures. Our capture of TB disease diagnoses may have been incomplete as well. TB reporting data was only available for cases reported to ADPH; thus cases diagnosed and treated out of state would not have been included. It seems highly likely, however, that treating physicians of patients on TNFi would have been notified of such an event given the extent of patient education regarding infection risk while on TNFi and the potential severity of TB disease should it have occurred. There may have been undiagnosed Alabama TB cases in both TNFi recipients and in the general pediatric population, particularly since use of immunosuppressive therapies may result in false negative TST results. A complete failure to diagnose TB disease seems unlikely given the potential severity of TB disease in children especially in the presence of immunosuppression. A delay in TB diagnosis beyond the study period was also possible but any delays would be at least partially mitigated by the duration of the study for nearly eight years. There is also a possibility of ascertainment bias, as clinicians may be more likely to suspect and evaluate for TB in a child on a TNFi. Given the disease severity of this particular case, it is likely that the diagnosis would have been reached regardless of prior TNFi usage. Missed or delayed diagnoses in the general population, thus increasing the measured risk associated with use of TNFi, is also possible, however this seems unlikely again due to the severity of progressive tuberculosis in childhood with prolonged time to diagnosis. Since TB is reportable by law, this should have minimized failure to capture any diagnosed TB.

An important strength of this study is our unique ability to capture the vast majority of pediatric TNFi usage in the state of Alabama. Additionally, usage of the state database to identify all diagnosed cases of TB disease, as well as manual review of all diagnosed cases of TB to screen for TNFi use, provides additional certainty to the data reported herein. The minimal use of TNFi not captured by the EMR at CoA likely had negligible statistical influence on the results.

As many as 40% of children with complex underlying medical conditions who develop TB disease may not be identified by current TB screening recommendations developed for otherwise healthy children [11]. With proper screening for TB infection or disease at the time of diagnosis of inflammatory conditions that require use of immune-modulating therapies, some cases of latent TB infection might be identified and the risk of progression to active disease decreased. Current treatment guidelines for JIA and IBD in children do not specify how to identify the patient on TNFi with increased risk of TB infection who might benefit from ongoing TB screening and testing while on TNFi therapy [8, 12]. However ongoing assessment of TB exposure risks is essential for this population and at least annual screening using a validated questionnaire to assess TB risk factors in combination with laboratory testing if risk factors are identified is one approach to risk assessment [13, 14]. The drawbacks and benefits of laboratory testing for TB infection, including discomfort and the costs associated with screening tests as well as additional costs of evaluating false positive findings, should be considered by the physicians in light of the local risks of TB exposure.

Continued efforts to educate families on the importance of reporting contact with persons with TB infection or disease might also help to decrease the risks of TB disease. Better screening tests for TB infection and disease are needed, especially in children and adolescents treated with biologic response modifiers, both to minimize the risk of missing TB infection and disease and to avoid unnecessary TNFi treatment delays and interruptions associated with false positive results.

## Conclusion

The incidence of TB infection was rare among children treated with TNFi. However, the incidence was higher than the general rate in Alabama, so continued vigilance is warranted, particularly among patients with additional risk factors including but not limited to active inflammatory disease, its treatments, and potential exposures to infectious tuberculosis.

## Abbreviations

ADPH: Alabama department of public health
CoA: Children’s of Alabama
EMR: Electronic medical record
IBD: Inflammatory bowel disease
JIA: Juvenile idiopathic arthritis
RA: Rheumatoid arthritis
TB: Tuberculosis
TNFi: Tumor necrosis factor inhibitor
TST: Tubcerculin skin test
